# Downregulation of protein kinase C-α enhances intracellular survival of Mycobacteria: role of PknG

**DOI:** 10.1186/1471-2180-9-271

**Published:** 2009-12-24

**Authors:** Shivendra K Chaurasiya, Kishore K Srivastava

**Affiliations:** 1 Division of Microbiology, Central Drug Research Institute, Lucknow, 226001, India

## Abstract

**Background:**

Intracellular trafficking of mycobacteria is comprehensively dependent on the unusual regulation of host proteins. Recently, we have reported that infection of macrophages by *Mycobacterium tuberculosis *H37Rv (Rv) selectively downregulates the expression of PKCα while infection by *Mycobacterium smegmatis *(MS) does not.

**Results:**

Based on our earlier study, we have extrapolated for the first time that knockdown of PKCα, impairs phagocytosis of mycobacteria by macrophages while their intracellular survival is drastically increased. *Mycobacterium bovis *BCG (BCG) and *Mycobacterium tuberculosis *H37Ra (Ra) have also been shown to downregulate the expression of PKCα during the infection. Since PknG is uniquely expressed in BCG, Ra, Rv but not in MS and has been reported to promote intracellular survival of mycobacteria, led us to believe that PknG may be involved in such downregulation of PKCα. THP-1 cells infected with recombinant MS expressing PknG (MS-G), showed significant reduction in PKCα expression. In normal THP-1 cells survival of MS-G was enhanced as compared to MS, while their behavior in PKCα deficient cells could not be distinguished. The results strongly demonstrate that pathogenic mycobacteria recognize and then inhibit PKCα to circumvent phagocytosis and the hostile environment of macrophages. We emphasize that, this inhibition is controlled by PknG.

**Conclusions:**

All together, our data reveal a mechanism that shows substantial interdependence of PKCα with PknG, in sustaining mycobacterial infection.

## Background

*Mycobacterium tuberculosis *(Mtb), the causative agent of tuberculosis, has infected billions of people worldwide. Phagocytic cells are critical for host defense against infection by capturing invading pathogens and killing them inside the bactericidal milieu of lysosomes as well as in processing and presenting the pathogen derived antigens. Based on the ability to infect and cause diseases, mycobacteria can be classified into species that cause TB in humans or in animals, including Mtb and *M. bovis*, and species that are generally non-pathogenic, such as MS and *M. vaccae*. The survival of pathogenic mycobacteria within macrophages involves the inhibition of several host cell processes which allow them unlike non-pathogenic species to survive inside host cells. Host processes manipulated by pathogenic mycobacteria include fusion of phagosomes with lysosomes, acidification of phagosomes and resistance to killing by oxygenated metabolites. Antigen presentation, apoptosis and the stimulation of bactericidal responses due to the activation of pathways involving mitogen-activated protein kinases (MAPKs), interferon-γ (IFN-γ) and calcium (Ca^2+^) signaling are also inhibited. The phagocytosis of pathogen is associated with an increase in cellular Ca^2+ ^and subsequent activation of Ca^2+ ^dependent events leading to destruction of invading bacilli [1]. Pathogenic mycobacteria inhibit the Ca^2+ ^flux which is usually associated with phagocytosis [2,3]. Ca^2+ ^is required for the activation of certain isoforms of PKC and the calmodulin kinase pathways, which are both potential upstream activators of MAP kinases [4].

Modulation of host cellular pathways may be influenced by signal transduction molecules expressed by pathogenic bacteria. The Mtb genome encodes 11 eukaryotic-like serine/threonine kinases [5,6]. Various signal-transduction pathways utilize protein phosphorylation/dephosphorylation in regulating different cellular activities such as adaptation and differentiation, immune response and cell division. Several studies have shown that macrophages infected with pathogenic mycobacteria show reduced activation of MAP kinases as compared with non-pathogenic mycobacteria resulting in the decreased production of NOS_2 _and TNF-α in infected macrophages [7,8].

Recent studies have highlighted the role of protein kinases in the biology and pathogenesis of mycobacteria. PknG, a cytosolic protein of Mtb, increases intracellular survival by inhibiting the fusion of mycobacterial phagosome with lysosome. Deletion of this gene in BCG results in the lysosomal localization of mycobacteria. Likewise MS expressing recombinant PknG is able to prevent the fusion of phagosome with lysosome [9]. The members of the PKC-family of proteins are classified in three groups, based on the mechanisms regulating their activation in response to different stimuli [10,11]. PKC has been implicated in various macrophage functions like phagocytosis, maturation of phagosome, immunity to infection, apoptosis and the productions of cytokines/chemokines/immune effector molecules [10,12-14]. PKC-α regulates phagocytosis and the biogenesis of phagolysosome by promoting the interaction of phagosome with late endososme and lysosomes [13,15-17]. PKC-α also plays important role in the killing of intracellular pathogens [14], however its role in mycobacterial pathogenesis has never been described. In our earlier study, we have shown that macrophages infected with Rv show decreased expression of PKC-α as compared to macrophages infected with MS, suggesting that difference in the intracellular survival of pathogenic and non-pathogenic mycobacteria may be related to their ability to downregulate PKC-α [18]. In present study, to understand the role of PKC-α in survival or killing of mycobacteria within macrophages, we selectively knocked-down PKC-α of macrophages and examined their ability to kill intracellular bacilli. We are first to report the (1) decrease in phagocytosis of mycobacteria by PKC-α deficient macrophages (2) knockdown of PKC-α results in increased survival of mycobacteria within macrophages (3) PknG from Mtb selectively downregulates PKC-α during infection (4) Expression of PknG in MS reduces the phagocytosis by macrophages and (5) the downregulation of PKC-α is mainly due to the proteolytic degradation by PknG.

## Results

### Downregulation of macrophage specific PKC-α by mycobacteria

**Figure 1:**
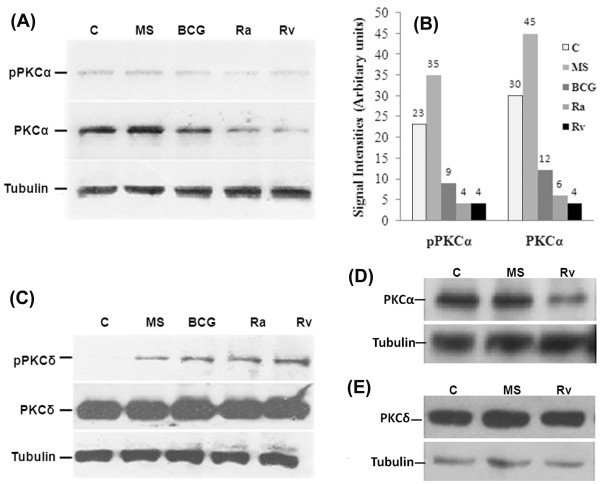
**Downregulation of PKC-α expression by mycobacteria**. THP-1 cells were incubated for 4 h in the presence of mycobacteria (MOI = 1:20) as indicated (C, uninfetced). The cells were lysed, and equal amounts of total cell lysates (20 μg) were resolved by SDS-PAGE and immunoblotted with an antibody against **(A) **PKC-α and phosphorylated form of PKC-α (Thr^638^), **(B) **Densitometric analysis of PKC-α and pPKC-α blots shown in fig. 1A, **(C) **PKC-δ and phospho-PKCδ (Thr^505^). The lower parts of the blots were probed with an anti-tubulin antibody, to assure equal protein loading (lower panel), **(D) **and **(E) **level of PKC-α and PKC-δ in mouse peritoneal macrophages. Each experiment was repeated at least 3 times.

Previous studies suggest that Rv, Ra and BCG are less efficiently taken up by macrophages as compared to MS [19] and have the ability to survive and multiply within macrophages. Infection of Rv but not MS inhibits macrophage PKC-α. The novel (PKC-δ and PKC-θ) and conventional (PKC-ζ) isoforms are not down regulated by Rv infection of macrophages [18]. To know whether infection of macrophages with BCG and Ra also results in the downregulation of PKC-α, we infected macrophages with mycobacteria and observed that infection of THP-1 cells with BCG and Ra also decreased the expression (2.5 and 5.7 fold respectively) as well as the phosphorylation of PKC-α by 2.5 and 5 fold respectively (Fig. 1A and 1B). Regulation PKC-δ was similar by MS, BCG, Ra and Rv (Fig. 1C) suggesting that pathogenic mycobacteria selectively downregulate PKC-α. The downregulation of PKC-α was also evident in primary mouse peritoneal macrophages when incubated with Rv (Fig. 1D and 1E).

### Decreased phagocytosis and increased survival of BCG and MS within PKC-α deficient THP-1 cells

**Figure 2:**
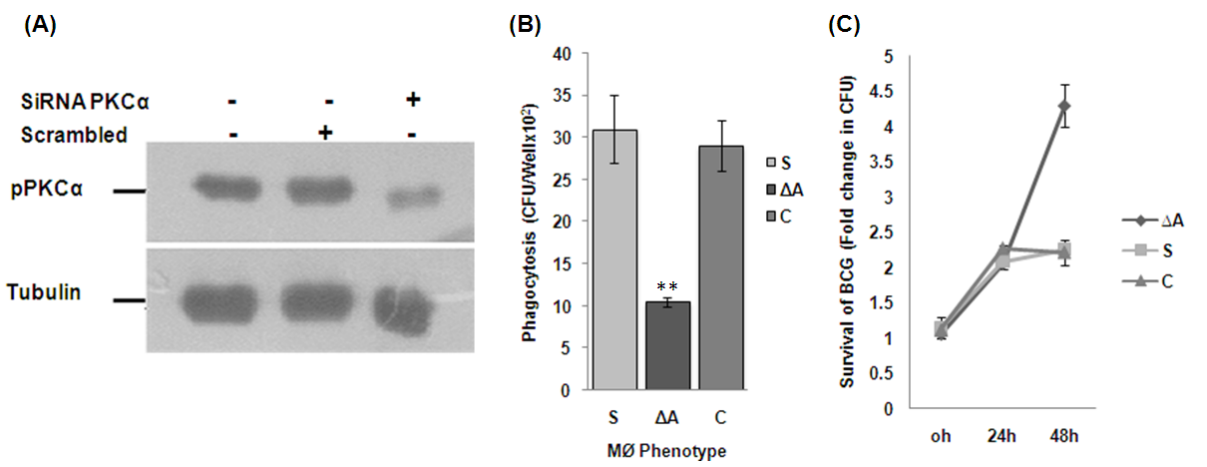
**Phagocytosis and survival of BCG in PKC-α deficient THP-1 cells**. THP-1 cells were incubated in the presence of 30 nM PMA for 24 h. Then cells were transfected with 20 nM SiRNA and level of PKC-α were determined by immunoblotting. (A) 24 h after transfection, level of PKC-α and PKC-δ in cells transfected with SiRNA targeting PKC-α or scrambled SiRNA, (B) 24 h after transfection, (ΔA) cells transfected with SiRNA targeting PKC-α and (S) cells transfected with scrambled SiRNA and control cells (C) were infected with BCG (MOI = 1:10) for 2 h, washed and remaining extracellular bacilli were killed by amikacin treatment for 1 h and lysed in 0.05% SDS and plated. Colony forming units (cfu) were determined after 4 week of incubation. Tukey (T) test was performed for statistical analysis of data (C) Survival of BCG in THP-1 cells transfected with either SiRNA targeting PKC-α (ΔA) or scrambled SiRNA (S) after 24 and 48 h, since phagocytosis of BCG in control and PKC-α deficient cells was different, CFU at 0 h was considered 1 and survival of BCG is presented as fold increase in the number of cfu as compared to the initial phagocytosis. Data are means ± standard deviations from three independent experiments each performed in 4 replicates. (** = p < 0.005).

Our initial study has proven that regulation of macrophage PKC-α by mycobacteria is species dependent [18]. To study the effect of PKC-α knockdown on the survival/killing of mycobacteria, THP-1 cells were transfected with SiRNA targeting PKC-α. SiRNA specifically reduced the expression of PKC-α by 70-90% (Fig. 2A). Infection of PKC-α deficient cells resulted in the significant (p < 0.005) reduction in phagocytosis of BCG. Data show that phagocytosis of BCG by PKC-α deficient cells was 2.8 fold reduced when compared to control (Fig. 2B). Intracellular survival of BCG was found to be increased in PKC-α deficient cells. Since phagocytosis of bacilli by normal and by PKC-α deficient cells was different, we presented the survival of BCG as fold increase in the number of intracellular bacilli as compared to the initial phagocytosis (Fig. 2C). The specificity of PKC-α SiRNA was confirmed by transfecting mouse macrophage cell line, J774A.1 and showing that SiRNA blocked PKC-α, only in THP-1 cells (data not shown).

**Figure 3:**
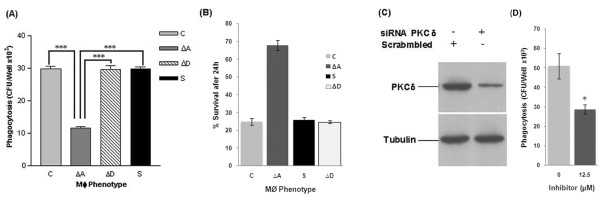
**Phagocytosis and survival of MS in PKC-α deficient THP-1 cells**. THP-1 cells were incubated in the presence of 30 nM PMA for 24 h. Then cells were transfected with 20 nM SiRNA and after 24 h level of PKC were determined by immunoblotting. (A) 24 h after transfection control cells (C) and (ΔA) cells transfected with SiRNA PKCα, (ΔD) cells transfected with SiRNA PKCδ, (S) cells transfected with scrambled SiRNA (PKC-α SiRNA which does not block PKCα), were infected with MS (MOI = 1:10) for 2 h, washed and remaining extracellular bacilli were killed by amikacin treatment for 1 h, again washed, lysed in 0.05% SDS and plated for cfu. 'T' test was performed for statistical analysis of data, (B) 24 h after infection % survival of MS in THP-1 cells transfected with either SiRNA targeting PKC-α (ΔA) or scrambled SiRNA (S), because phagocytosis of MS was different in control and PKC-α deficient cells, cfu at 0 h was considered 100% and survival of MS is presented as percentage of the initial cfu that survive in macrophages after 24 h. (C) 24 h after transfection, level of PKC-δ in cells transfected with SiRNA targeting PKC-δ or scrambled SiRNA, (D) Phagocytosis of MS by mouse macrophage cell line J774A.1 cells pretreated with an inhibitor of PKC-α (Go6976) for 30 minute before infection. Data are means ± standard deviations from three independent experiments each performed in 4 replicates. (*** = p < 0.0001, * = p < 0.05).

To clearly understand the specific role of PKC-α in the phagocytosis and survival of mycobacteria, we used MS (which does not downregulate PKC-α) for infection. Knockdown of PKC-α resulted in the significant (p < 0.0001) decrease in the phagocytosis of MS by macrophages (Fig. 3A). Results show that phagocytosis of MS is 2.6 fold less in PKC-α deficient cells as compared to normal cells. Inhibition of phagocytosis was specific to the inhibition of PKC-α as knockdown of PKC-δ did not inhibit the phagocytosis or survival (Fig. 3A, 3B and 3C). When survival of MS in macrophages deficient in PKC-α was compared with normal cells, we found that survival of MS was increased in the PKC-α deficient macrophages. Since phagocytosis of MS by normal and PKC-α deficient cells was different, we expressed intracellular survival of MS as percentage of the initial bacilli uptake. In normal macrophages, only 25% of initial bacilli survived as contrast to 65% survival in PKC-α deficient cells (Fig. 3B). The results were confirmed with J774A.1 cells using Go6976 (inhibitor of PKC-α) which represented similar level of inhibition in phagocytosis (Fig. 3D).

### Detection of expression of PknG in different mycobacteria

PknG has been shown to inhibit phagosomal maturation [9], a process that is promoted by PKC-α [13,15-17], and which helps in survival of mycobacteria within macrophages. There seems to be an inverse relationship between PknG and PKC-α in terms of regulation of events involved in phagosomal maturation and intracellular survival of mycobacteria. This led us to think about some relationship between PknG and PKC-α in determining the intracellular survival of mycobacteria. To check the expression of PknG in mycobacteria, we cloned, expressed, purified protein [see additional file 1] and raised antiserum. Immunoblotting of mycobacterial lysates using anti-PknG serum shows that PknG is expressed in Rv, Ra and BCG but not in MS [see additional file 1(C)].

### Construction of recombinant MS expressing PknG

To underline the specific role of PknG in controlling PKC-α, the gene was expressed in MS. Cloning of *pknG *in pMV361 vector was confirmed by restriction digestion [see additional file 1(D)]. For expression, pMV361-*pknG *was electroporated into MS and resultant clones (MS-G) were confirmed by PCR [see additional file 1(E)] and immunoblotting using anti-PknG serum [see additional file 1(F)].

### Recombinant MS downregulates macrophage PKC-α during infection

**Figure 4:**
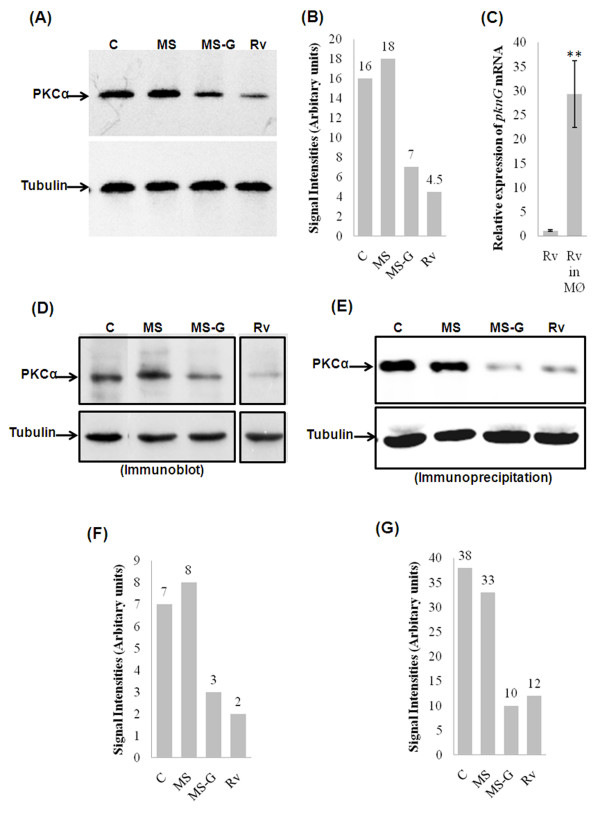
**Downregulation of expression of macrophage PKC-α by recombinant mycobacteria expressing PknG**. (A) The THP-1 cells infected with either wild type or recombinant mycobacteria were lysed, and equal amounts of total cell lysates (20 μg) were resolved by SDS-PAGE and immunoblotted with an antibody against PKCα. The lower parts of the blots were probed with an anti-tubulin antibody, to assure equal protein loading, (B) Densitometric analysis of blots shown in fig. 5A, (C) THP-1 cells infected with Rv were osmotically lysed and bacteria were recovered by centrifugation and total bacterial RNA was isolated. Total RNA was also isolated from bacterial suspension in RPMI-1640 medium which was used for infection of THP-1 cells. RNA samples were treated with DNAse I and cDNA were prepared using random hexamer primers and was used as template for Cyber Green real time PCR using *pknG *specific primers (values presented are normalized against *16S rRNA*), Data are means ± standard deviations from five independent experiments each performed in 3 replicates. (** = p < 0.005). (D) experiment identical to 5A was performed with J774A.1 cells, (E) equal amounts of total cell lysates of J774A.1 cells infected with mycobacteria were immunoprecipitated with anti-PKC-α antibody and level of PKC-α was analyzed by immunoblotting. Same amounts of lysates were also immunoprecipitated with anti-tubulin antibody to serve as control, (F) Densitometric analysis of blots shown in fig. 5D, (G) Densitometric analysis of blots shown in fig.5E. The experiments were repeated at least 3 times.

BCG and Ra are laboratory produced avirulent strains that still infect and grow within mammalian hosts, though they do not lead to the chronic disease that their virulent counterparts do. However, BCG and Ra are able to inhibit the maturation of phagosome which is consistent with their ability to downregulate PKC-α. PknG is expressed by Rv, BCG, and Ra (bacilli are able to downregulate PKC-α) but PknG is not expressed in MS (does not downregulate PKC-α). This led us to speculate that PknG might contribute to the downregulation of PKC-α by mycobacteria and resulting in the increased intracellular survival. To test this hypothesis, we infected THP-1 cells with MS-G and studied the level of macrophage PKC-α. We found that THP-1 cells infected with MS-G show 2.2 and 2.5 fold decreased level of PKC-α when compared to control cells and cells infected with MS respectively (Fig. 4A and 4B). In the same experiment, expression of *pknG *mRNA in Rv was found to be increased by 32 fold (Fig. 4C). Similar results were observed with J774A.1 cells. Immunoprecipitation (Fig. 4E, 4G) as well as western blot analyses (Fig. 4D, 4F) of lysates from J774A.1 cells infected with mycobacteria confirmed downregulation of PKC-α by MS-G.

### Expression of PknG in MS mimics the effect of PKC-α knockdown

**Figure 5:**
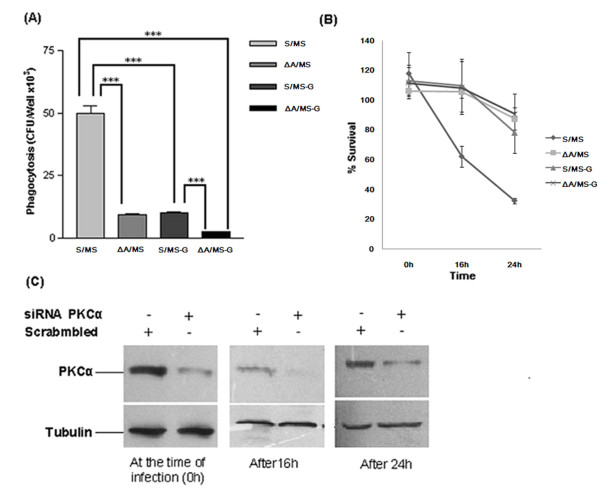
**Comparison of phagocytosis and intracellular survival of MS and MS-G in normal and in PKC-α deficient THP-1 cells**. (A) THP-1 cells were incubated in the presence of 30 nM PMA for 24 h. Cells were then transfected either with SiRNA targeting PKC-α (ΔA) or scrambled SiRNA (S) and after 24 h were infected with MS or MS-G (MOI = 1:10) for 2 h, washed and remaining extracellular bacilli were killed by amikacin treatment for 1 h, again washed and internalized bacteria were released by lysis of macrophages with 0.05% SDS and plated then cfu were counted, (S/MS) phagocytosis of MS by normal THP-1 cells, (ΔA/MS) phagocytosis of MS by PKC-α deficient THP-1 cells, (S/MS-G) phagocytosis of MS-G by normal THP-1 cells, (ΔA/MS-G) phagocytosis of MS-G by PKC-α deficient THP-1 cells. 'T' test was performed for statistical analysis of data. (B) % survival of MS and MS-G in normal and PKC-α deficient THP-1 cells. Because, phagocytosis of MS and MS-G were different in control and in PKC-α deficient cells, cfu at 0 h was considered 100% and survival of MS is presented as percentage of the initial cfu. (C) At each time point of experiment, level of PKC-α in cells transfected either with SiRNA targeting PKC-α or scrambled SiRNA was also determined by immunoblotting, to confirm the levels of PKC-α throughout the experiment. Data are means ± standard deviations from three independent experiments each performed in 4 replicates. (*** = p < 0.0001).

PknG down regulates PKC-α, resulting in the inhibition of phagocytosis and increased survival of mycobacteria within macrophages. This raised the possibility of impaired phagocytosis of MS-G in comparison to MS. To test this we infected THP-1 cells with MS and MS-G and compared the phagocytosis. We observed significantly reduced (5 fold less) phagocytosis of MS-G (p < 0.0001), which was comparable with the phagocytosis of MS by PKC-α deficient THP-1 cells (Fig. 5A). When phagocytosis of MS-G by normal and by PKC-α deficient macrophages was compared, 4 fold decrease (p < 0.0001) in phagocytosis of MS-G by PKC-α deficient cells was observed (Fig. 5A). In the same experiment, we also compared the survival of MS-G and MS in normal and in PKC-α deficient macrophages. We observed that survival of MS-G in normal macrophages was higher than MS but in PKC-α deficient macrophages, MS and MS-G survived equally which was higher than the survival of MS in normal macrophages (Fig. 5B). Western blotting of samples at each time point confirmed the knockdown of PKC-α throughout the experiment (Fig. 5C).

### Direct inhibition of PKC-α by PknG

**Figure 6:**
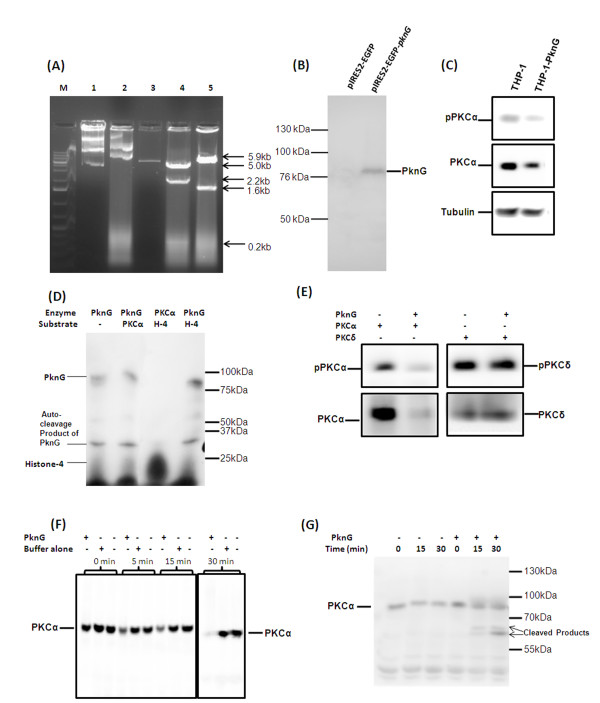
**Mechanism of downregulation of PKC-α by PknG**. (A) Cloning of *pknG *in pIRES2-EGFP vector; M, DNA ladder; 1, pIRES2-EGFP-*pknG *undigested; 2, pIRES2-EGFP undigested; 3, pIRES2-EGFP digested with *Bam*HI; 4, pIRES2-EGFP-*pknG *digested with *Hind*III; 5, pIRES2-EGFP-*pknG *digested with *Bam*HI, right oriented recombinants will produce 1.6 kb fragment; (B) and (C) pIRES2-EGFP-*pknG *was transfected in THP-1 cells and after 48 h cells were lysed and immunoblotted with anti-serum against PknG and with PKC-α antibodies, lane 1 macrophages transfected with vector alone and lane 2 transfected with pIRES2-EGFP-*pknG*. (D) 5 μg PknG was incubated with immunoprecipitated PKC-α in kinase buffer for 30 min in presence of [γ32P]-ATP then resolved by 8% SDS-PAGE and exposed to X-Ray film., lane 1 PknG alone; lane 2 PKC-α and PknG, lane 3 PKC-α and Histone-4 and lane 4 PknG and Histone-4. (E) THP-1 cell lysate was immunoprecipitated with either antibodies against PKC-α or PKC-δ using protein G Sepharose. The immunoprecipitated proteins were incubated with 5 μg purified PknG for 1 h and immunoblotted with PKC-α and PKC-δ antibodies. (F) Macrophage cell lysate (50 μg) was incubated with 5 μg purified PknG or buffer alone for indicated times and immunoblotted with PKC-α antibodies. (G) THP-1 cell lysate was immunoprecipitated with antibodies against PKC-α and immunoprecipitates were treated with 5 μg of purified PknG for the time points as indicated and then immunoblotted with PKC-α antibody. The experiments were repeated at least 3 times.

PknG expressing mycobacteria are able to downregulate the expression of PKC-α. Whether downregulation of PKC-α require mere presence of PknG during infection or PknG regulate some cellular process which results in downregulation PKC-α. Cellular process/target which is responsible for downregulation of PKC-α may be of mycobacterial or host origin. To explore whether PknG alone or with mycobacteria is required for the downregulation of PKC-α, *pknG *was cloned in pIRES2-EGFP vector (Fig. 6A) and pIRES2-EGFP-*pknG *was transfected into THP-1 cells. Expression of PknG in transfected cells was confirmed by western blotting (Fig. 6B). Expression of PknG in THP-1 cells resulted in the decreased level of PKC-α (Fig. 6C) suggesting that mere expression of PknG in macrophages without mycobacteria downregulates PKC-α. This also suggest that downregulation of PKC-α involves modulation of some host process. In order to dissect, whether this effect of PknG is a direct interaction or pathway mediated, we performed kinase activity of PknG. PknG undergoes autophosphorylation (Fig. 6D, lane 1, 92 kDa band) and phosphorylates it's self cleavage product (Fig. 6D, lane 1, 32 kDa band) but does not phosphorylate PKC-α (Fig. 6D, lane 2) or histone (Fig. 6D, lane 4). PKC-α phosphorylates histones (Fig. 6D, lane 3, 25 kDa band) which confirms that PKC-α used in assay was active. To test if there is any possibility that PknG dephosphorylates PKC-α, the immunoprecipitated PKC-α (contain adequate amount of phosphorylated form PKC-α too) was treated with purified PknG. To our surprise, levels of PKC-α and phosphorylated PKC-α were reduced upon treatment with PknG suggesting degradation of PKC-α (Fig. 6E). This also suggests that the observed reduced level of phosphorylated form in earlier experiments was due to decrease in total PKC-α protein. However, PknG treatment did not affected PKC-δ (which is used as control in the experiment) confirming the specificity of PknG for PKC-α rather than general protease activity (Fig. 6E). For better understanding of the direct effect of PknG on PKCα, we incubated macrophage lysate with purified PknG and observed degradation of PKC-α (Fig. 6F). To further look for the degradation of PKC-α in a time dependent manner, we treated purified PKC-α with PknG. The immunoblotting with PKC-α antibody showed that PknG cleaves PKC-α proteolytically and the resulting product was detectable with anti-PKC-α antibody (Fig. 6G).

## Discussion

The induction of various macrophage functional responses such as the oxidative burst, MHC class II protein expression, interleukin 1-β production, tumoricidal activity, and phagocytosis are thought to be regulated at least in part via PKC dependent signaling [10]. PKC regulates IgG mediated phagocytosis by human macrophages and is reported to translocate to the membrane before significant ingestion takes place. PKC inhibitors decreased phagocytosis in a dose dependent manner. Phagosomal localization of PKC also increases during phagocytosis [12]. PKC-α promote Fc-γ receptor mediated phagocytosis and signal transduction and inhibition of PKC-α results in inhibition of phagocytosis [20]. During phagocytosis, MARCKS, PKC-α and Myosin 1 are recruited along with F-actin and talin in the cortical cytoplasm adjacent to forming phagocytic cups. After completion of particle ingestion, myosin I, F-actin, and talin dissociate from phagosomes. By contrast, MARCKS and PKC-α remain associated with the phagosome membrane until after acquisition of the lysosomal marker LAMP-1. Phagocytosis results in rapid and sustained phosphorylation of MARCKS, suggesting PKC-α dependent phosphorylation is an early signal required for zymosan phagocytosis and that MARCKS and PKC-α have roles in phagosome maturation [16]. PKC-α has also been shown to promote phagosomal maturation by regulating the association of LAMP-1 and flottilin-1 on phagosomal membrane and inhibition of PKC-α results in the impairment of phagosomal maturation [15]. When tubercular and non-tubercular bacilli interact with macrophages, PKC isoforms are regulated in different manner. We were first to report that Rv and MS activate and phosphorylate novel PKC isoforms. PKC-α (a conventional isoform) was downregulated by Rv but not by MS [18]. It was reported that macrophages derived from BCG resistant and BCG sensitive mice differ in their PKC activity and that macrophages from BCG resistant mice show increased PKC activity as compared to macrophages from BCG sensitive mice [21]. In present study our main objective has been to decipher the role of PKC-α in mycobacterial survival/killing. Knockdown of PKC-α resulted in the decreased phagocytosis of BCG and MS by macrophages while their intracellular survival was increased (Fig. 2B, 2C, 3A, 3B). Inhibition of PKC-δ did not affect phagocytosis or survival of MS (Fig. 3A and 3C). These data show important role of PKC-α in phagocytosis as well as in killing of mycobacteria and suggest that downregulation of PKC-α during infection is a strategy utilized by pathogenic mycobacteria which help them to avoid the lysosomal machinery and survive inside host cells. This idea is further supported by the observation that BCG, Ra, and Rv (bacilli can multiply within macrophages) can downregulate PKC-α while MS does not (Fig. 1A and 1B). Previous studies with other organisms have also emphasized the role of PKC-α in phagocytosis and killing of pathogens. Encapsulated *Streptococcus suis *can survive and multiply inside macrophages while non-encapsulated *S. suis *does not. Infection of J774A.1 macrophages with the non-encapsulated mutant of *S. suis *results in the enhanced activation of PKC-α, whereas the encapsulated strain showed reduced activation of PKC-α resulting in the reduced phagocytosis of bacteria [22]. Inhibition of PKC-α by *Leishmania donovani *lipophosphoglycan results in the decreased phagocytosis by murine macrophages as well as impaired recruitment of LAMP-1 on the phagosomal membrane resulting in the arrest of phagosomal maturation [13,23]. Survival of *L. donovani *promastigotes also involves inhibition of PKC-α. Intracellular survival of a *L. donovani *mutant defective in lipophosphoglycan repeating units synthesis, which normally is rapidly degraded in phagolysosomes, was enhanced in DN PKC-α-over-expressing RAW 264.7 cells [13-15,23]. Interestingly, a recent study has identified two Mtb strains (*i.e*. HN885 and HN1554) among a bank of clinical isolates showing defect in phagocytosis when compared to strain Erdman. Despite reduced phagocytosis, ingested bacilli replicated at a faster rate than strain Erdman [24]. These observations suggest that clinical spectrum of pathogenic mycobacteria also include strains capable of avoiding phagocytosis. Saprophytic and opportunistic pathogenic mycobacteria are more readily ingested than are the members of the Mtb family [19]. Inhibition of PKC-α by BCG, RA and Rv but not by MS (Fig. 1A and 1B) suggests that difference in the uptake and intracellular survival of pathogenic and non-pathogenic mycobacteria is related at least in part, to their ability to downregulate PKC-α. Interestingly, mammalian PKC-α has similarity with mycobacterial PknG [25]. PknG has been shown to promote intracellular survival of mycobacteria by inhibiting the process of phagosomal maturation. PknG is secreted into the cytosol of infected macrophage suggesting the possibility that it may access host cell molecules. There is impaired recruitment of LAMP-1 on phagosomes containing live mycobacteria expressing PknG [9]. Phagosomes containing live pathogenic mycobacteria actively retain Coronin 1, which is generally released prior to fusion with lysosome [26]. In a further study, Coronin 1 was shown to be required for activation of Ca^2+ ^dependent phosphatase calcineurin, thereby blocking the lysososmal delivery of mycobacteria [27]. PKC-α has been shown to phosphorylate p57 (human homologue of coronin family actin-binding protein) and PKC mediated phosphorylation of p57 is required for its dissociation from phagosomes as well as for recruitment of LAMP-1 to the phagosomes, an event necessary for the fusion of phagosomes with lysosomes [17]. PknG is expressed in BCG, Ra and Rv but not in MS [see additional file 1(C)] as referred earlier too [28], led us to speculate that PknG enhances survival of mycobacteria by inhibiting PKC-α. When macrophages were infected with MS-G, expression of PKC-α was decreased as compared to uninfected and MS infected macrophages (Fig. 4A, 4B, 4D, 4E, 4F and 4G) confirming that PknG directs the downregulation of PKC-α by mycobacteria which supports our hypothesis that PknG mediated enhanced intracellular survival of mycobacteria involves inhibition of PKC-α. During Rv infection, the levels of *pknG *transcripts were increased by 32 fold as compared to extracellular mycobacteria (Fig. 4C) which reiterates their ability to affect mycobacterial survival. In normal macrophages phagocytosis of MS-G was reduced in comparison to MS, which was similar with the reduced phagocytosis of MS by PKC-α deficient macrophages as compared to normal macrophages (Fig. 5A). Phagocytosis of MS-G was further reduced in PKC-α deficient macrophages (Fig. 5A) suggesting that, once MS starts expressing PknG the behavior of MS-G, in terms of phagocytosis look similar in pattern with BCG (Fig. 6A). Moreover, survival of MS-G in normal macrophages mimics the survival of MS in PKC-α deficient macrophages which was higher than the survival of MS in normal macrophages (Fig. 5B). MS-G survives equally in normal and in PKC-α deficient macrophages (Fig. 5B). These observations further support the view that intracellular survival of mycobacteria involves the inhibition of PKC-α by mycobacterial PknG. Expression of PKC-α was decreased in macrophages expressing PknG (Fig. 6B and 6C) confirming that PknG mediated inhibition of PKC-α involves alteration with host cell pathway rather than mycobacterial pathway. PknG may modulate the host cell processes by phosphorylation of host cell molecule. In a study, level of PKC-α was shown to be decreased by phosphorylation/dephosphorylation resulting in the degradation of PKC-α suggesting that phosphorylation/dephosphorylation is also linked with the degradation of PKC-α [29]. Thus PknG may contribute to the downregulation of PKC-α by directly phosphorylating it. PknG neither phosphorylated (Fig. 6D) nor dephosphorylated PKC-α (Fig. 6E) neglecting the possibility of involvement of phosphorylation/dephosphorylation mediated pathway in downregulation of PKC-α. Surprisingly, incubation of PKC-α but not PKC-δ with PknG resulted in the degradation of PKC-α (Fig. 6E). Besides auto-phosphorylation [30,31], PknG is reported to catalyse self cleavage [31] which suggests the possibility of proteolytic degradation of PKC-α by PknG. PKC-δ was unaffected by PknG confirming the specificity of PknG for PKC-α. Incubation of macrophage lysate with PknG also resulted in specific degradation of PKC-α which further supports that PknG mediated downregulation of PKC-α may be direct and possibly does not require host or mycobacterial mediators (Fig. 6F). When immunoprecipitated PKC-α was incubated with PknG, PKC-α was specifically degraded by PknG treatment (Fig. 6G) and this degradation may be due to the cleavage of PKC-α by PknG treatment as evident by detection of a low molecular weight protein by anti-PKC-α antibody (Fig. 6G). However, this cleavage product of PKC-α was not evident in earlier experiments when macrophages were infected with mycobacteria. We speculated that this could be either due to the lower level/less accumulation of PknG (and degradation product) in macrophages as compared to exogenous addition or may be the further degradation of cleaved products within the cell. Therefore when the proteins were incubated in higher amounts the cleavage product could be seen. Thus we concluded that the presence of PknG in macrophages either with mycobacteria or as a protein, precisely control PKC-α. Moreover, when pathogenic mycobacteria reside in macrophages it raises the level of PknG [Fig. 4C] which strengthens the understanding that more inactivation of PKC-α may be possible. Hence, this study is first to report that PknG downregulates PKC-α and their association discriminates the fates of pathogenic and nonpathogenic mycobacteria in macrophages. Recently, *L. donovani *GP63 has been shown to proteolytically cleave many host proteins resulting in the inactivation of MAPKs [32] suggesting that cleavage of host proteins is a defense strategy utilized by intracellular pathogens.

During tuberculosis, host defense may be determined, in part, by the capacity of macrophages to bind and ingest Mtb. Phagocytosis by macrophages represents the early step of the mycobacterial infection. It is governed both by the nature of the host receptors used and the ligands exposed on the bacteria as well as the environment where host cells encounter mycobacteria [[24,25], and [33-36]]. Final outcome of the infection is determined by cumulative effect of all these factors.

## Conclusions

Expression of PknG in BCG, Ra and Rv but not in MS represents an interesting example of evolution where pathogen has developed strategies for modulation of host molecule to avoid uptake and killing by the entities designed for their killing. Interestingly, PknG directs the downregulation of PKCα and further negotiating the uptake and survival of mycobacteria. Our data clearly and for the first time reveal that pathogenic mycobacteria downregulates PKCα predominantly to avoid phagocytosis and killing by macrophages. Detailed understanding of the events leading to the downregulation of PKCα by PknG inside host cells open a new chapter which may further project to the identification of new therapeutic targets for mycobacterial infections.

## Methods

### Reagents

Antibodies against PKCs and phospho-PKCs were purchased through Santa Cruz Biotech Inc. and Cell Signaling Technologies, USA, respectively. Horseradish peroxidase-linked secondary antibodies, polyvinylidene difluoride membrane, RPMI-1640, DMEM, HEPES, sodium bicarbonate, Imidazole, IPTG and Protein G Sepharose beads were purchased from Sigma chemicals. Enhanced chemiluminescence kit (ECL) was from GE Healthcare. Middlebrook 7H9 medium and endotoxin-free fetal calf serum (FCS) were purchased from Difco laboratories (Sparks, MD). SiRNAs were procured through Ambion. SiRNA transfection reagent was purchased from Bio-Rad (USA). Cell Line Nucleofector Kit V was purchased from Amaxa Inc. USA.

### Cell culture

The THP-1 human macrophage-like cell line was acquired from the American Type Culture Collection, USA and cultured in RPMI-1640 medium containing 2 mM L-glutamine, 10 mM HEPES, 1 mM sodium pyruvate, 4.5 g/L glucose, 1.5 g/L sodium bicarbonate, supplemented with 10% heat inactivated fetal calf serum and 0.05 mM β-mercaptoethanol at 37°C, 5% CO_2_. Cells were treated with 30 nM PMA for 24 h before using for the experiments. The J774A.1 murine macrophage cell line was maintained at 37°C, 5% CO_2 _in DMEM containing 10% fetal calf serum, 2 mM glutamine and essential amino acids.

### Mycobacteria and macrophage Infection

*Mycobacterium tuberculosis *H37Rv (Rv), *Mycobacterium tuberculosis *H37Ra (Ra), *Mycobacterium bovis *BCG (BCG) and *Mycobacterium smegmatis *MC^2 ^155 (MS) were grown in Middlebrook (MB) 7H9 medium supplemented with 0.5% glycerol, ADC supplement, 0.5% BSA, fraction V, 0.2% dextrose, 0.85% NaCl and 0.05% Tween 80. Cultures were incubated at 37°C. Mycobacteria grown in mid-log phase were used for infecting THP-1 cells. The bacterial suspension was washed and resuspended in RPMI-1640 containing 10% FCS. Bacterial clumps were disaggregated by vortexing five times (each cycle~2 min) with 3-mm sterile glass beads, and then passed through 26 gauge needle 10 times to disaggregate any remaining clumps. The total number of bacilli per milliliter of suspension was ascertained by measuring OD at 650 nm and by further counting for cfu on MB7H10 agar plates.

### Infection and preparation of cell lysates for western blotting

THP-1 cells were seeded at 2 × 10^6 ^cells/well in 6 well plates and were subsequently incubated with 20, mycobacteria/macrophage, for 4 h and lysed in phosphorylation buffer as described previously [18]. Alternatively, 2 × 10^6 ^peritoneal macrophages from BALB/c mouse were also infected with MS and Rv. Total 20 μg protein sample was analyzed by 10% SDS-PAGE and electroblotted as described previously [18]. Briefly, after blocking, the membranes were incubated overnight at 4°C with antibodies (anti PKC-α and anti PKCδ, 1:1000, anti pPKC-α and anti pPKCδ, 1:1000, anti tubulin, 1:5000, anti PknG, 1:1000) in 0.1% TBST containing 3% BSA, with gentle shaking. After four washes with 0.05% TBST, the membrane was incubated with goat anti-rabbit (anti-mouse when detecting tubulin) polyclonal antibodies conjugated to horseradish peroxidase (1:50000) in 0.1%TBST containing 3% BSA for 1 h at room temperature. After four washes with 0.05% TBST, the blots were developed using ECL reagents and were analyzed on Chemi-Doc XRS system (Bio-Rad Laboratories, Hercules, CA) using Quantity One program.

### Cloning, expression and purification of PknG

Rv genomic DNA was used as a template for amplification of *pknG *gene by PCR. The gene was cloned in either pTriEx4 or in pMV361 vectors using the primers containing the desired restriction enzyme sites (Table 1). For expression in *E. coli*, *pknG *with *Hind*III flanking sites was subcloned in pTriEx4 vector. For expression in MS, *pknG *with *Eco*RI/*Hind*III flanking sites was subcloned into pMV361 vector. For expression in THP-1 cells, *pKnG *cloned in pTriEx4 vector was digested with *Eco*RI and *Xho*I and ligated to pIRES2-EGFP vector predigested with *EcoR*I and *Sal*I. Cloning and orientation of gene were confirmed by PCR and restriction digestion. *E. coli *BL21 (DE3) cells were transformed with pTriEX4-*pknG *and transformants were grown in LB medium containing ampicillin (100 μg/ml) at 37°C, till OD at 600 nm reached 0.6. IPTG was then added to a final concentration of 0.8 mM and cultures were further grown for an additional 4 h at 37°C with shaking. Cells were harvested by centrifugation at 5000 × g for 15 min and resuspended in binding buffer [Sodium Phosphate 20 mM (pH 7.4), NaCl 50 mM, Imidazole 5 mM, PMSF 1 mM] and sonicated on ice for 2 min. After sonication TritonX-100 was added in cell lysate at a final concentration of 1% before centrifugation at 30000 × g for 30 min at 4°C. Supernatant was loaded onto Ni^2+^-NTA column, washed with 60 mM Imidazole and 6-His-PknG was eluted with 200 mM Imidazole. Affinity purified 6-His-PknG was further purified by size exclusion chromatography using Sephacryl 200 column and AKTA Prime protein purification system (GE healthcare).

**Table 1:** List of PCR primers used in the study.

Primers	Genes	Description
CCCAAGCTTATGGCCAAAGCGTCAGAGAC	*pknG*	Forward with *Hind*III site, for pTriEx4 vector

CCCAAGCTTTTAGAACGTGCTGGTGGGCC	*pknG*	Reverse with *Hind*III site, for pTriEx4 and pMV361 vector

CCC GAA TTC ATG GCC AAA GCG TCA GAG AC	*pknG*	Forward with *EcoR*1 site, for pMV361 vector

TCAAACGCAGCAAGGGTCAGAAAC	*pknG*	Forward, for real time PCR

TCGTTGTAGACCAAGCCGATGGAA	*pknG*	Reverse, for real time PCR

TGCAAGTCGAACGGAAAGGTCTCT	*16S rRNA*	Forward, for real time PCR

AGTTTCCCAGGCTTATCCCGAAGT	*16S rRNA*	Reverse, for real time PCR

For expression in MS, cells were transformed with pMV361-*pknG *and grown in MB7H9 medium supplemented with Kanamycin (25 μg/ml).

For raising antiserum, purified 6-His-PknG chimeric protein was injected subcutaneously with Freund's incomplete adjuvant. Immunization was performed on days 0, 7 and 21. On day 30 rabbit was bled and the serum was separated. The antiserum was confirmed for its reactivity with PknG protein using western blotting and ELISA.

### Knockdown of PKC-α

THP-1 cells were seeded at a density of 2 × 10^6 ^per well in 6 well tissue culture plate 24 h before transfection. The medium was replaced at the time of transfection. Cells were transfected with 20 nM SiRNA using 3 μl transfection reagent in 1.25 ml medium. After 4 h an additional 1 ml of fresh medium was added to each well and incubated for 24 h. After transfection viability of monolayers was monitored by the trypan blue dye exclusion method. An aliquot of the cultures were confirmed for the knockdowns of PKC-α and PKC-δ by western blotting.

### Transfection of THP-1 cells with pknG

THP-1 cells were transfected with pIRES2-EGFP-*pknG *using Cell Line Nucleofector Kit V as per manufacturer's protocol. Transfection was confirmed by fluorescent microscopy as well as by western blotting using anti-PknG serum.

### Assay for phagocytosis and intracellular survival of mycobacteria

24 h post transfection cells were washed and infected with mycobacteria to give a multiplicity of infection (MOI) of 10. Cells were incubated at 37°C and 5% CO_2 _for 2 h and then washed 3 times with incomplete medium to remove most of the extracellular bacteria. Cultures were further incubated in complete medium supplemented with Amikacin (200 μg/ml) for 1 h at 37°C and 5% CO_2_. At 0, 16, 24 and 48 h cells were washed 3 times with PBS and lysed (Before lysis the viability of the monolayer was monitored by the trypan blue dye exclusion method in all of the experiments described) with 0.05% SDS solution and serially diluted in 7H9 medium with 0.05% Tween-80, and plated onto 7H10 agar plates containing 10% OADC. Plates were supplemented with Kanamycin (25 μg/ml) where required. CFU were counted after incubation at 37°C for 4 to 5 days for MS and 3-4 weeks for BCG.

### Quantitation of RNA during infection

To isolate RNA from intracellular mycobacteria, macrophages were subjected to osmotic lysis and released bacteria were pelleted and total RNA was isolated using Tri-Reagent (MRL) according to manufacturer's instruction. Total RNA (4 μg) was digested with RNAse free DNAse and used for the synthesis of cDNA with random hexamer primers using Revertaid H Minus First Strand cDNA Synthesis Kit (Fermentas). Quantitative real time PCR was performed in 96 well plate on Light Cycler 480 system (Roche) using QuntiTect Cyber green PCR mix (Qiagen) and results were analyzed using Light Cycler 480 software (Roche). Primer pairs used for amplification of *pknG *and *16s rRNA *(internal control for *pknG*) are listed in Table 1.

### Immunoprecipitation of PKC

Protein G Sepharose beads were washed twice with PBS and were incubated with 4 μg of polyclonal anti-PKC antibodies per 100 μl of beads for 1 h at room temperature. After washing twice with PBS equal amounts (approximately 1 mg) of total cell lysates were incubated with 200 μl of beads for overnight in cold. After incubation beads were washed with PBS.

### Phosphorylation and dephosphorylation assays for PKC-α by PknG

To look if there is any effect on PKC-α by PknG, radioactive kinase assay was performed using [γ32P]-ATP and PKC-α as substrate as described previously [11,37].

## Authors' contributions

KKS supervised the research. KKS and SKC performed experiments, analyzed data, prepared and approved the final manuscript.

## Supplementary Material

Additional file 1 **Cloning, expression, purification and immunodetection of PknG**. (A) Cloning of *pknG *in pTriEx4 vector; M, 500 bp DNA ladder; 1, pTriEx4-*pknG *digested with *Bam*HI, right oriented recombinants will produce 0.7 kb fragment; 2, pTriEx4-*pknG *digested with *Hind*III, recombinants will produce 2.2 kb fragment; 3, pTriEx4 vector digested with *Hind*III; 4, pTriEx4-*pknG *undigested, (B) overexpression and purification of PknG; 1, cells transformed with vector; 2, cells transformed with recombinant; 3, cells transformed with vector and induced with IPTG; 4, cells transformed with recombinant and induced with IPTG; 5 and 6, purified PknG. (C) Immunodetection of PknG in mycobacteria; equal amounts of total cell lysates (20 μg) were resolved by SDS-PAGE and immunoblotted with polyclonal antiserum against PknG (1) MS (2) BCG (3) Ra (4) Rv (D) Cloning of *pknG *in pMV361 vector; M, 500 bp DNA ladder; 1, pMV361 vector uncut; 2, pMV361-*pknG *uncut; 3, pMV361 digested with *EcoR*I and *Hind*III; 4, pMV361-*pknG *digested with *Eco*RI and *Hind*III; (E) PCR of *pknG *from genomic DNA; M, 1 kb DNA ladder; 1, MS; 2, MS-pMV361-*pknG*; (F) expression of PknG in MS; equal amounts of total cell lysates (20 μg) were resolved by SDS-PAGE and immunoblotted with polyclonal antiserum against PknG, (1) MS-pMV361 (2) MS-pMV361-*pknG *(3) MS and (4) Rv. Click here for file
