# Supplementary figures and images for: Downregulation of protein kinase C-α enhances intracellular survival of Mycobacteria: role of PknG
Source: BMC Microbiol. 2009 Dec 24;9:271. doi: 10.1186/1471-2180-9-271 (PMC2816201; doi:10.1186/1471-2180-9-271)

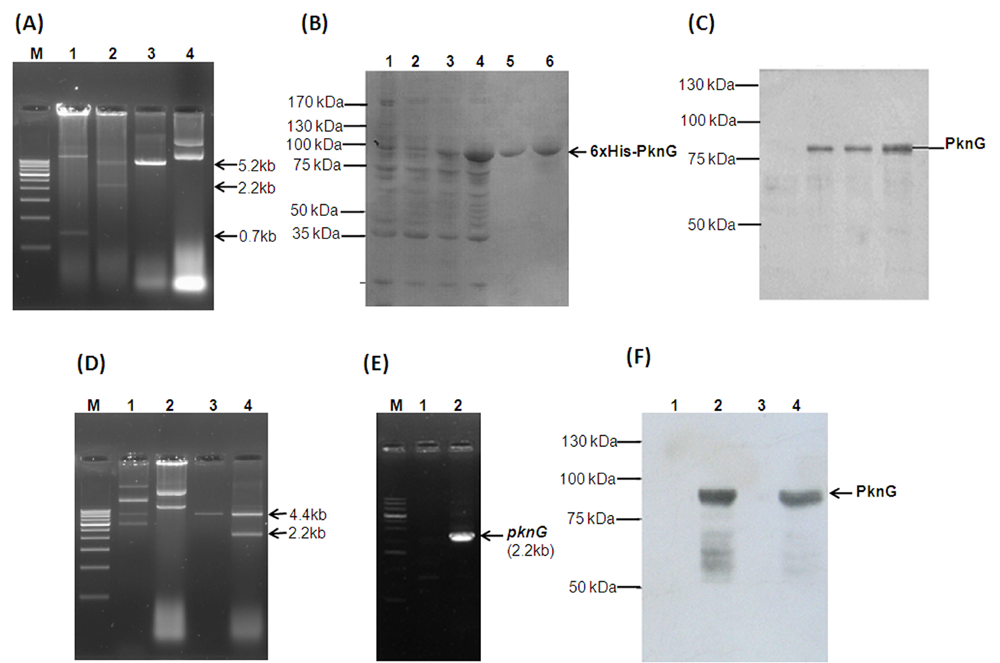

Supplement: Additional file 1 — Cloning, expression, purification and immunodetection of PknG. (A) Cloning of pknG in pTriEx4 vector; M, 500 bp DNA ladder; 1, pTriEx4-pknG digested with BamHI, right oriented recombinants will produce 0.7 kb fragment; 2, pTriEx4-pknG digested with HindIII, recombinants will produce 2.2 kb fragment; 3, pTriEx4 vector digested with HindIII; 4, pTriEx4-pknG undigested, (B) overexpression and purification of PknG; 1, cells transformed with vector; 2, cells transformed with recombinant; 3, cells transformed with vector and induced with IPTG; 4, cells transformed with recombinant and induced with IPTG; 5 and 6, purified PknG. (C) Immunodetection of PknG in mycobacteria; equal amounts of total cell lysates (20 μg) were resolved by SDS-PAGE and immunoblotted with polyclonal antiserum against PknG (1) MS (2) BCG (3) Ra (4) Rv (D) Cloning of pknG in pMV361 vector; M, 500 bp DNA ladder; 1, pMV361 vector uncut; 2, pMV361-pknG uncut; 3, pMV361 digested with EcoRI and HindIII; 4, pMV361-pknG digested with EcoRI and HindIII; (E) PCR of pknG from genomic DNA; M, 1 kb DNA ladder; 1, MS; 2, MS-pMV361-pknG; (F) expression of PknG in MS; equal amounts of total cell lysates (20 μg) were resolved by SDS-PAGE and immunoblotted with polyclonal antiserum against PknG, (1) MS-pMV361 (2) MS-pMV361-pknG (3) MS and (4) Rv. [file 1471-2180-9-271-S1.TIFF]
